# Bioinformatics-based analysis of the roles of sex hormone receptors in endometriosis development

**DOI:** 10.7150/ijms.79516

**Published:** 2023-02-05

**Authors:** Xiaoling Zhao, Weimin Kong, Chunxiao Zhou, Boer Deng, He Zhang, Huimin Guo, Shuning Chen, Zhendong Pan

**Affiliations:** 1 Department of Gynecologic Oncology, Beijing Obstetrics and Gynecology Hospital, Capital Medical University, Beijing Maternal and Child Health Care Hospital, Beijing, China.; 2 Division of Gynecologic Oncology and Lineberger Comprehensive Cancer Center, University of North Carolina at Chapel Hill, Chapel Hill, NC, USA.

**Keywords:** Endometriosis, Hormone receptor, Bioinformatics analysis

## Abstract

Endometriosis is a hormone-dependent disease in women of reproductive age and seriously affects women's health. To analyze the involvement of sex hormone receptors in endometriosis development, we performed bioinformatics analysis using four datasets derived from the Gene Expression Omnibus (GEO) database, which may help us understand the mechanisms by which the sex hormones act in vivo in endometriosis patients. The enrichment analysis and protein-protein interaction (PPI) analysis of the differentially expressed genes (DEGs) revealed that there are different key genes and pathways involved in eutopic endometrium aberrations of endometriosis patients and endometriotic lesions, and sex hormone receptors, including androgen receptor (AR), progesterone receptor (PGR) and estrogen receptor 1 (ESR1), may play important roles in endometriosis development. Androgen receptor (AR), as the hub gene of endometrial aberrations in endometriotic patients, showed positive expression in the main cell types for endometriosis development, and its decreased expression in the endometrium of endometriotic patients was also confirmed by immunohistochemistry (IHC). The nomogram model established based on it displayed good predictive value.

## Introduction

Endometriosis is caused by the presence of endometrium-like tissues outside of the uterus 1. It is estimated that endometriosis can affect 10-15% of reproductive-age women, resulting in pelvic pain and infertility 1, 2. The long-term presence of endometriosis also carries the risk of cancers 3, 4. Several hypotheses, such as retrograde menstruation theory, have been proposed to explain the etiology and pathogenesis of endometriosis, however, none of them can fully explain it.

It is now basically clear that endometriosis is a hormone-dependent inflammatory disease 5. In the endometrium of patients with endometriosis, estrogen, which can promote endometrial cell proliferation and inflammation, was dominant while progesterone was resistant and failed to properly antagonize the effects of estrogen 5, 6. The progesterone resistance and estrogen dominance in ectopic lesions lead to increased lesion growth and contribute to pelvic pain and infertility 5, 6. Androgen, which can reduce the chronic pain and inflammation, can be converted to estrogen by aromatase in the eutopic and ectopic endometrium of women with endometriosis, thereby increasing local estrogen levels 7. So far, the exact mechanisms by which these hormones act in vivo remain unclear, making prevention and treatment challenging.

**Figure 1:**
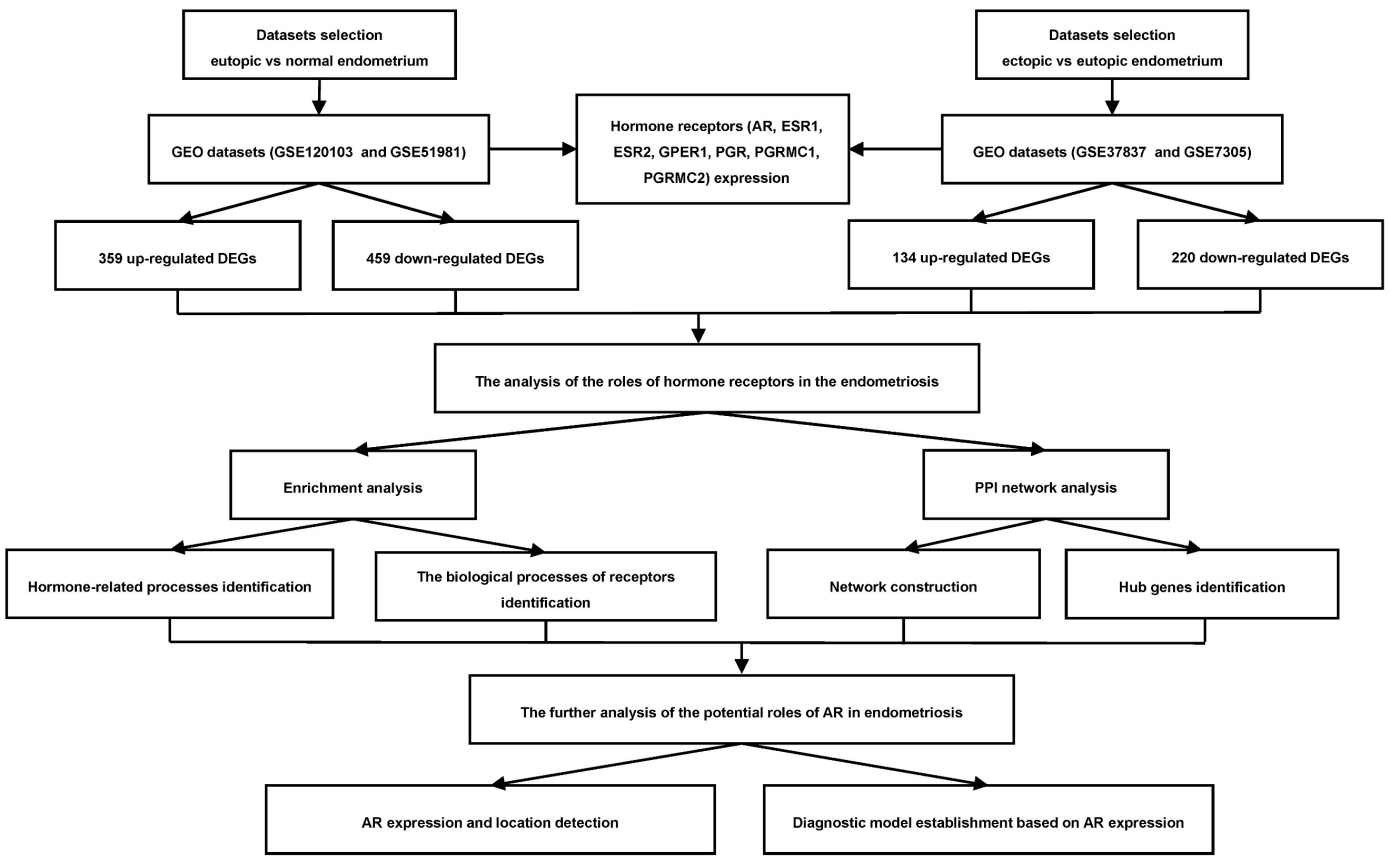
Flowchart of the integrated analysis for endometriosis. AR, androgen receptor; ESR1, estrogen receptor 1; ESR2, estrogen receptor 2; GPER1, G-protein coupled estrogen receptor 1; PGR, progesterone receptor; PGRMC1, progesterone receptor membrane component 1; PGRMC2, progesterone receptor membrane component 2; GEO, Gene Expression Omnibus; DEGs, the differentially expressed genes; PPI, protein-protein interaction.

Currently, with the rapid development of sequencing technology and the emergence of bioinformatics analysis and public databases, we can obtain a massive amount of gene information to explore the underlying molecular mechanisms of the disease 8. The sex hormones mediate the biological effects on endometrium by binding to their receptors, occurring at cell surface and in the nucleus 5, 9. Understanding the roles of these receptors in the pathogenesis of endometriosis may help us uncover the mechanisms of these sex hormones' actions. Therefore, in this study, we aimed to investigate the roles of sex hormone receptors in endometriosis development by bioinformatics analysis, which may provide us with new insights into the disease. The workflow of this study was presented in Figure 1.

## Methods

### Data collection and DEGs identification

The gene expression profiles associated with endometriosis were obtained from the GEO database, which is searched using 'endometriosis' and 'endometrioma' as keywords and restricts the source of tissues to 'homo sapiens'. The information of the selected datasets is displayed in Table 1. Then the datasets (GSE51981, GSE120103, GSE37837 and GSE7305) with complete clinical information and sufficient case numbers were selected for further analysis. GSE51981, based on the GPL570 platform, includes 34 normal endometria and 77 endometria from endometriosis patients 10. GSE120103, based on GPL6480, includes 18 normal endometria and 18 endometria from endometriosis patients 11. The datasets GSE37837 and GSE7305 were produced using the GPL6480 and GPL570 platforms, which contained 36 samples (18 ectopic lesions and 18 matched control endometria from the same patients) and 20 samples (10 ectopic lesions and 10 matched control endometria from the same patients), respectively 12, 13.

The DEGs were identified using GEO2R, an interactive tool that can compare two or more groups of samples with the limma package 14. After normalization and log2 transformation, the genes with a fold change >1 and a P value <0.05 were selected.

### Enrichment analysis

Enrichment analyses were performed using the Database for Annotation, Visualization and Integrated Discovery (DAVID) (https://david.ncifcrf.gov) 15. Biological processes (BP) were selected for further analysis to identify the biological attributes of the DEGs. A P value <0.05 was set as the cutoff for statistical significance.

### PPI network analysis

To explore the interactions among the identified DEGs, we mapped them to the Search Tool for the Retrieval of Interacting Genes/Proteins (STRING) database (https://cn.string-db.org) to assess the protein-protein interaction information 16. To ensure reliable interactions, only experiments were selected as the active interaction sources, with the minimum required interaction scores at 0.150. The other indicated network properties consist of organism (Homo sapiens); network type (full string network); and meaning of network edges (evidence). Then, Cytoscape software was used to visualize the PPI network 16. CytoHubba, a plug-in of Cytoscape software, was used to rank nodes and screen the hub genes 17. The top 10 genes calculated by topological algorithms were considered hub genes.

### The analysis of androgen receptor (AR) using the Human Protein Atlas (HPA) database

HPA (https://www.proteinatlas.org/) is a comprehensive database covering the protein expression of many cancerous and normal tissues, with millions of images for human tissue samples included 18, 19. The expression level of AR in human normal tissues was evaluated in the HPA database and presented as a histogram. The single-cell profiles of the endometrium are also pictured in the HPA database, and the expression level of AR in each cell type is presented.

### AR expression and location detection

Endometrial tissues and ectopic lesions were collected from patients with indications for hysterectomy in Beijing Obstetrics and Gynecology Hospital from January 2018 to December 2021. Normal endometria, including 3 postmenopausal endometria and 6 premenopausal endometria were collected from patients with grade III cervical intraepithelial neoplasia or stage IA1 cervical cancer. Ectopic lesions and matched eutopic endometrial samples were collected from 6 patients with endometriosis combined with grade III cervical intraepithelial neoplasia or cervical cancer stage IA1. All the patients had normal menstrual cycles and didn't receive any hormone therapy. This study was approved by the Ethics Committee of Beijing Obstetrics and Gynecology Hospital affiliated with Capital Medical University, and written informed consent was obtained from all patients.

Immunohistochemistry (IHC) was used to detect AR expression using an AR antibody (#DF6783, Affinity, Japan) at a dilution of 1:200. The H-score (H-score = [1*(% of cells 1+) + 2*(% of cells 2+) + 3*(% of cells 3+)], where 1 = weak expression, 2 = moderate expression, and 3 = strong expression) was applied to quantify the IHC images 20. Immunofluorescent (IF) staining was performed to determine the location of AR in the cells using an AR antibody (#DF6783, Affinity, Japan) at a dilution of 1:100.

### Identification of proteins that interact with hormone receptors

The interactors of hormone receptors were identified from two databases: GPS-Prot (http://www.gpsprot.org) and Biogrid (https://thebiogrid.org). GPS-Prot is a web-based visualization platform for PPIs that allows new user-generated data to be uploaded 21. Biogrid is a biomedical interaction repository with data compiled through comprehensive curation efforts 22. The differentially expressed interactors of AR between eutopic endometria and normal endometria with a fold change >1 and a P value <0.05 were selected for the establishment of the models.

### Diagnostic model establishment

A nomogram was established using the rms package in R software with GSE51981 as the training set 23. Genes included in the diagnostic model analysis were selected by least absolute shrinkage and selection operator (LASSO) regression using the glmnet package 24. Then, GSE120103, containing 36 samples, was used as the test set to verify the model. The receiver operating characteristic (ROC) curve calculated by the pROC package was used to test the efficacy of the diagnostic model 23.

## Results

### Identification of differentially expressed genes (DEGs) and sex hormone receptors expression

In the present study, a total of 6073 and 6633 DEGs between normal endometria and those of patients with endometriosis were identified in GSE120103 and GSE51981, respectively. 1787 DEGs were obtained from their intersection. Among them, the numbers of up-regulated genes and down-regulated genes in both two datasets were 359 and 459, respectively. A total of 2455 and 1835 DEGs between eutopic and ectopic endometrial tissues of the same patients from GSE37837 and GSE7305, respectively, were also selected. A total of 384 DEGs were found after intersection, comprising 134 up-regulated genes and 220 down-regulated genes in both two datasets. The genes were listed in Supplementary Table S1.

**Figure 2:**
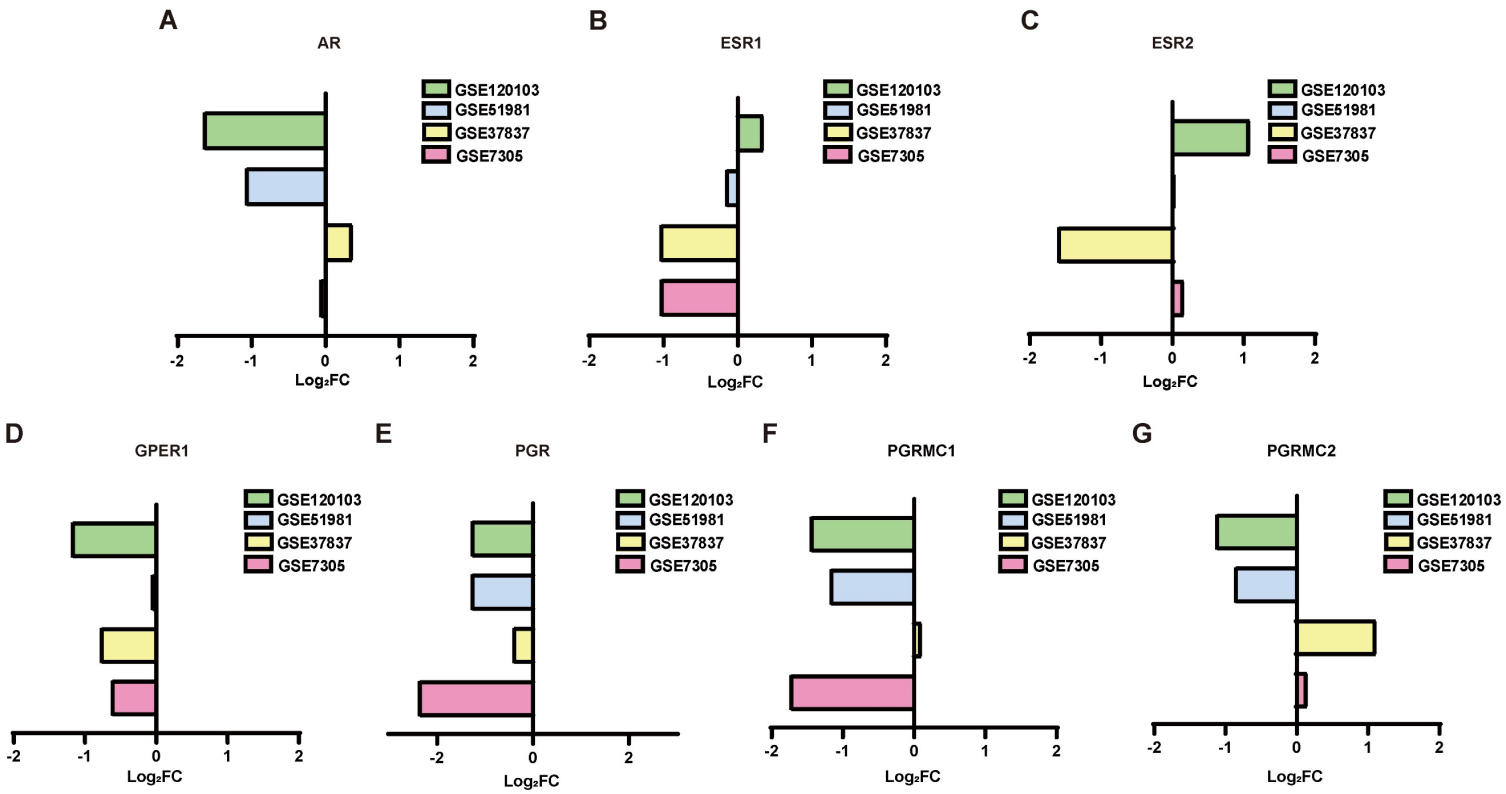
The expression of sex hormone receptors in the datasets. (A-G) The expression of sex hormone receptors, including AR (A), ESR1 (B), ESR2 (C), GPER1 (D), PGR (E), PGRMC1 (F) and PGRMC2 (G), in the datasets. AR, androgen receptor; ESR1, estrogen receptor 1; ESR2, estrogen receptor 2; GPER1, G-protein coupled estrogen receptor 1; PGR, progesterone receptor; PGRMC1, progesterone receptor membrane component 1; PGRMC2, progesterone receptor membrane component 2. FC of GSE51981 and GSE120103= the value of endometria from endometriosis patients/ the value of normal endometria; FC of GSE37837 and GSE7305= the value of ectopic endometria from the same patients/the value of eutopic endometria from endometriosis patients. FC, fold change.

Then the expression of sex hormone receptors in eutopic and ectopic endometria was analyzed in GSE51981, GSE120103, GSE7305 and GSE37837 (Figure 2A-G). In the eutopic endometria of endometriosis patients, AR, progesterone receptor (PGR) and progesterone receptor membrane component 1 (PGRMC1) showed decreased expression compared with normal endometria, with log_2_FC<1 in both the GSE51981 and GSE120103 datasets. In the ectopic endometria of endometriotic patients, only ESR1, one of the estrogen receptors, showed markedly decreased expression in both the GSE7305 and GSE37837 datasets, with log_2_FC<1.

### Enrichment analysis of the DEGs

To identify the biological functions of the DEGs in the development of endometriosis, enrichment analyses were conducted using DAVID, and the top 20 enriched biological processes were represented in Table 2-3. The DEGs between eutopic endometrium and normal endometrium mainly enriched in 'positive regulation of macromolecule metabolic process', 'positive regulation of metabolic process' and 'positive regulation of macromolecule biosynthetic process'. And the up-regulated genes in eutopic endometrium were mainly enriched in 'cell activation', 'anatomical structure development' and 'leukocyte activation' while the down-regulated genes were mainly enriched in 'chromosome organization', 'cellular component organization or biogenesis' and 'organelle organization'. For the DEGs between ectopic endometrium and eutopic endometrium, the genes were primarily enriched in 'anatomical structure morphogenesis', 'anatomical structure development' and 'single-multicellular organism process'. Moreover, the up-regulated genes in ectopic endometrium were significantly enriched in 'regulation of response to stimulus', 'regulation of multicellular organismal process' and 'positive regulation of response to stimulus', while the down-regulated genes were significantly enriched in 'regulation of cell cycle', 'cell cycle' and 'cell cycle process'.

Considering that endometriosis is a hormone-dependent gynecological disease, we screened the hormone-related biological processes. For eutopic endometrium derived from endometriotic patients and normal endometrium, the processes, including 'response to hormone', 'cellular response to hormone stimulus', 'response to steroid hormone', 'cellular response to peptide hormone stimulus', 'response to peptide hormone' and 'cellular response to steroid hormone stimulus', were screened. Both AR and PGR were involved in the biological processes named 'response to hormone', 'cellular response to hormone stimulus', 'response to steroid hormone' and 'cellular response to steroid hormone stimulus' (Table 4). For ectopic endometrium and eutopic endometrium of endometriotic patients, the biological processes, including 'cellular response to luteinizing hormone stimulus', 'response to luteinizing hormone', 'hormone catabolic process', 'response to peptide hormone', 'response to hormone', 'response to growth hormone', 'cellular response to peptide hormone stimulus' and 'hormone metabolic process', were screened. ESR1 involved in 'response to hormone' and 'hormone metabolic process' (Table 5).

In addition, we screened the biological processes in which sex hormone receptors were involved. The top 20 enriched biological processes were listed in Table 6. AR and PGR were involved in most of the processes in which the DEGs between eutopic endometrium and normal endometrium were significantly enriched. 'Positive regulation of macromolecule metabolic process', 'positive regulation of metabolic process' and 'positive regulation of macromolecule biosynthetic process' were the top 3 enriched processes in which both AR and PGR were involved. Only 15 significantly enriched biological processes in which PGRMC1 was involved. 'Single-organism cellular process', 'single-organism process' and 'cellular biosynthetic process' ranked top 3. ESR1 was also involved in almost all the enriched processes of the DEGs between ectopic endometrium and eutopic endometrium, and 'anatomical structure morphogenesis', 'anatomical structure development' and 'single-multicellular organism process' ranked the top 3.

### PPI network analysis of the DEGs

**Figure 3:**
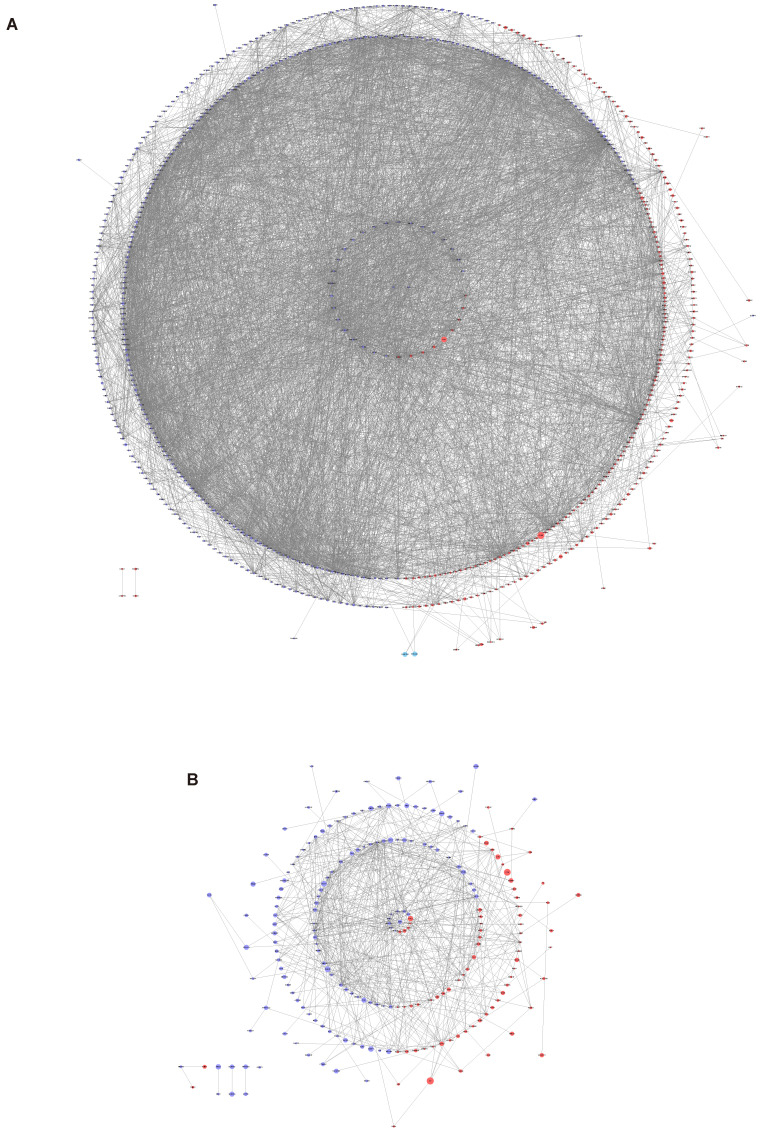
PPI network analysis of the DEGs. In the network, the red circles represent up-regulated genes, while the blue circles represent down-regulated genes. The size of the circle is positively correlated with |Log_2_FC|. (A) PPI network analysis of the DEGs between normal endometria and those of endometriosis patients. (B) PPI network analysis of the DEGs between eutopic endometria and ectopic endometria from the same patients. PPI, protein‑protein interaction.

To investigate the associations among the screened DEGs, PPI networks were constructed using Cytoscape software. And then the top 10 genes were selected from each method using CytoHubba. The network of the DEGs between normal endometrium and those of patients with endometriosis contained 683 nodes and 5105 edges, with AR, PGR and PGRMC1 involved in the network construction (Figure 3A). AR interacts with 9 up-regulated genes (KDM6B, SRC, FGR, NR4A1, FOS, CEBPB, TNK2, ZBTB16 and KAT5) and 25 down-regulated genes (NCOA4, SPOP, CTNNB1, SMARCA4, HSP90AB1, NCOR1, HSPA5, TCF4, HSP90AA1, KPNA3, TXNDC5, SMARCC1, DEPDC1, NUP107, APPL1, PIK3R1, ATRX, MAPK1, KDM4A, RAH, KDM5B, BECN1, KPNB1 and MYLIP) directly. PGR interacts with 3 up-regulated genes (KDM6B, SRC and NR4A1) and 6 down-regulated genes (MAPK1, NUP107, HSP90AA1, NCOR1, HSP90AB1 and SPOP) directly. PGRMC1 interacts with 1 up-regulated gene (HSD11B1L) and 5 down-regulated genes (PTPLAD1, MPRIP, HNRNPH, CANX and SRSI3) directly. Besides, the hub genes of the DEGs between eutopic endometrium of endometriosis patients and normal endometrium were calculated and displayed in Supplementary Table S2. Both AR and PGR were identified as the hub genes.

The network of the DEGs between ectopic endometrium and eutopic endometrium contained 213 nodes and 571 edges (Figure 3B). ESR1 interacts with 5 up-regulated genes (FHK2, ST13, CAV1, JUNB and EPAS1) and 8 down-regulated genes (SOX9, WHSC1, AURKA, XBP1, SMC2, MAP3K1, RAD51 and ZMYNDB) directly. What's more, ESR1 was also identified as one of the hub genes (Supplementary Table S2).

### The potential roles of AR in endometriosis development

The roles of estrogen and progesterone in endometriosis development and the relevance of their receptors to endometriosis have been discussed and validated in many studies 5, 6. Considering the lack of research on AR in endometriosis, we next explored the expression of AR in human tissues and cells to explore the possibility of AR involvement in the formation of endometriosis.

**Figure 4:**
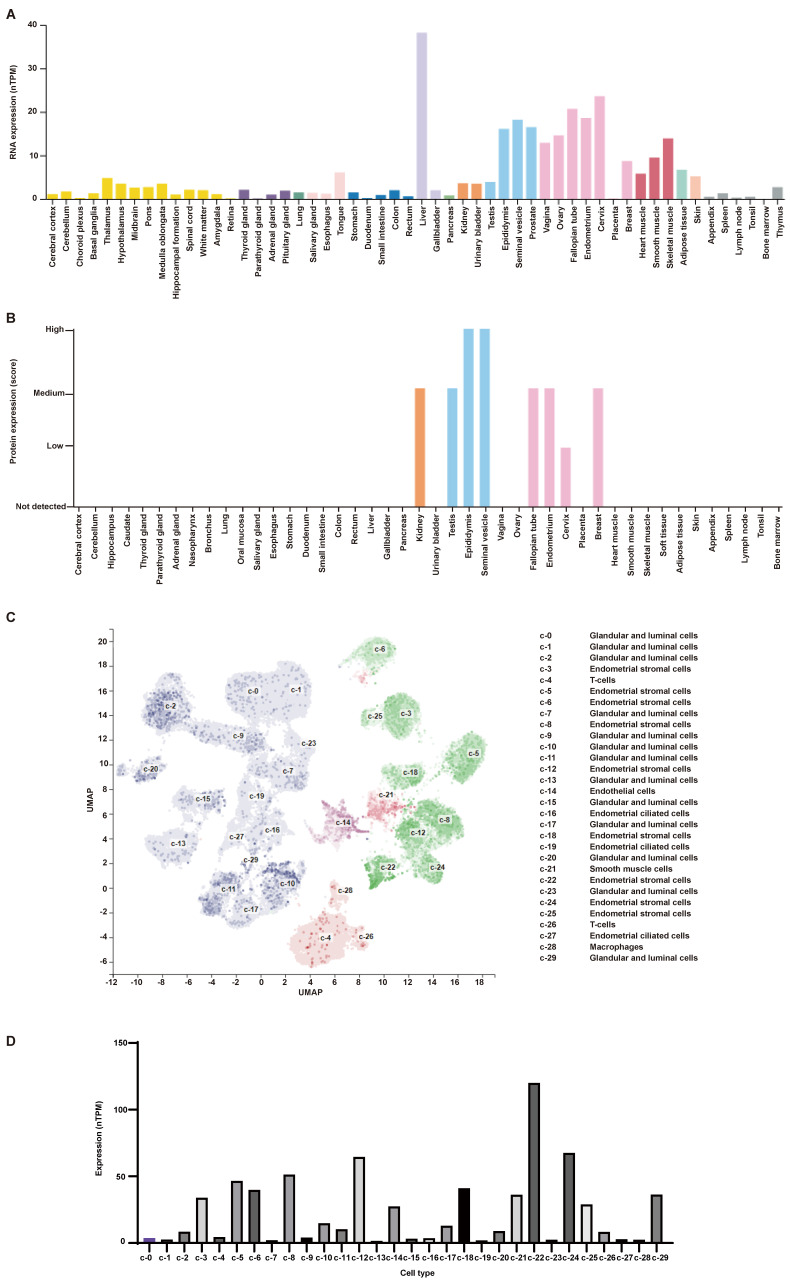
The expression of AR in human tissues obtained from HPA database. (A) The RNA expression level of AR in human tissues. (B) The protein expression level of AR in human tissues. (C-D) The single-cell profile of human endometrium (C) and AR expression in each cell type (D). AR, androgen receptor; HPA, the Human Protein Atlas.

**Figure 5:**
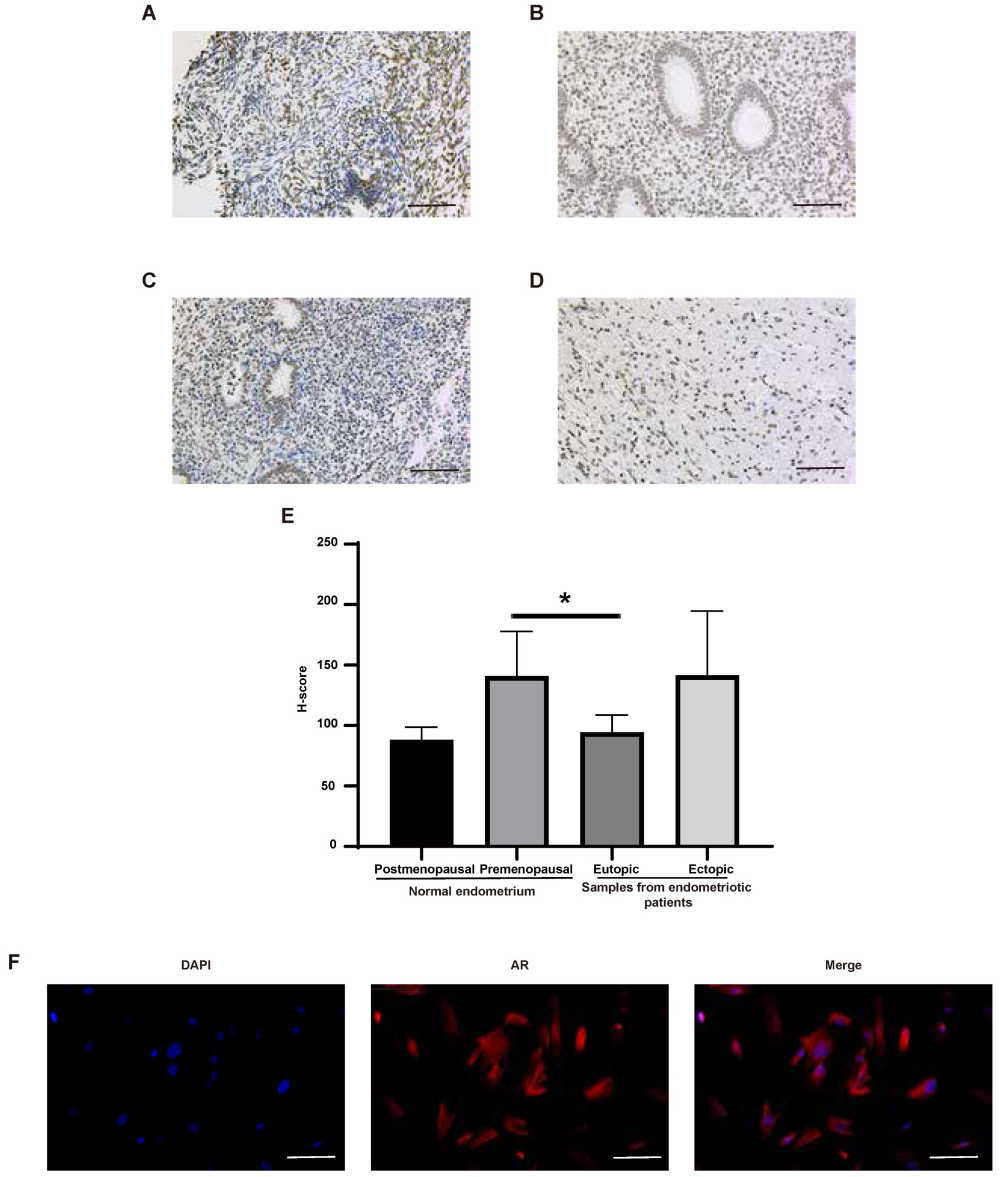
The expression of AR in human endometrial tissues. (A-D) Representative images of AR staining in postmenopausal endometrium (A), premenopausal endometrium (B), eutopic endometrium from endometriotic patients (C) and their matched ectopic lesion (D). (E) The corresponding histograms of positive AR staining level in different groups. (F) Representative images of AR staining in endometrial stromal cells. Scale bar 100 µm. AR, androgen receptor.

The expression of AR in normal human tissues was detected at both RNA and protein levels (Figure 4A-B). The results showed that the RNA expression of AR can be detected in all tissues except bone marrow, while its protein expression was found only in kidney, testis, epididymis, seminal vesicle, fallopian tube, endometrium, cervix and breast. What's more, AR is expressed in all cell types of endometria, with the greatest expression in endometrial stromal cells (Figure 4C-D). The expression of AR in endometrium tissues was also detected in this study using the IHC method (Figure 5A-E). We found that AR expression is retained in the post-menopausal endometrium and positive AR staining can be detected in both endometrial epithelial cells and stromal cells using the IHC method. The expression level of AR in normal premenopausal endometria was significantly higher than that in endometria derived from endometriotic patients (P<0.05). No significant difference in AR expression was found between eutopic endometria and their matched ectopic lesions.

Endometrial stromal cells are the main cell type expressing AR in endometrial tissues, and they are also an important cell type for endometriosis development 25. So, we examined the location of AR in endometrial stromal cells using the IF method. The results showed that AR localizes to the cytosol and nucleus (Figure 5F).

**Figure 6:**
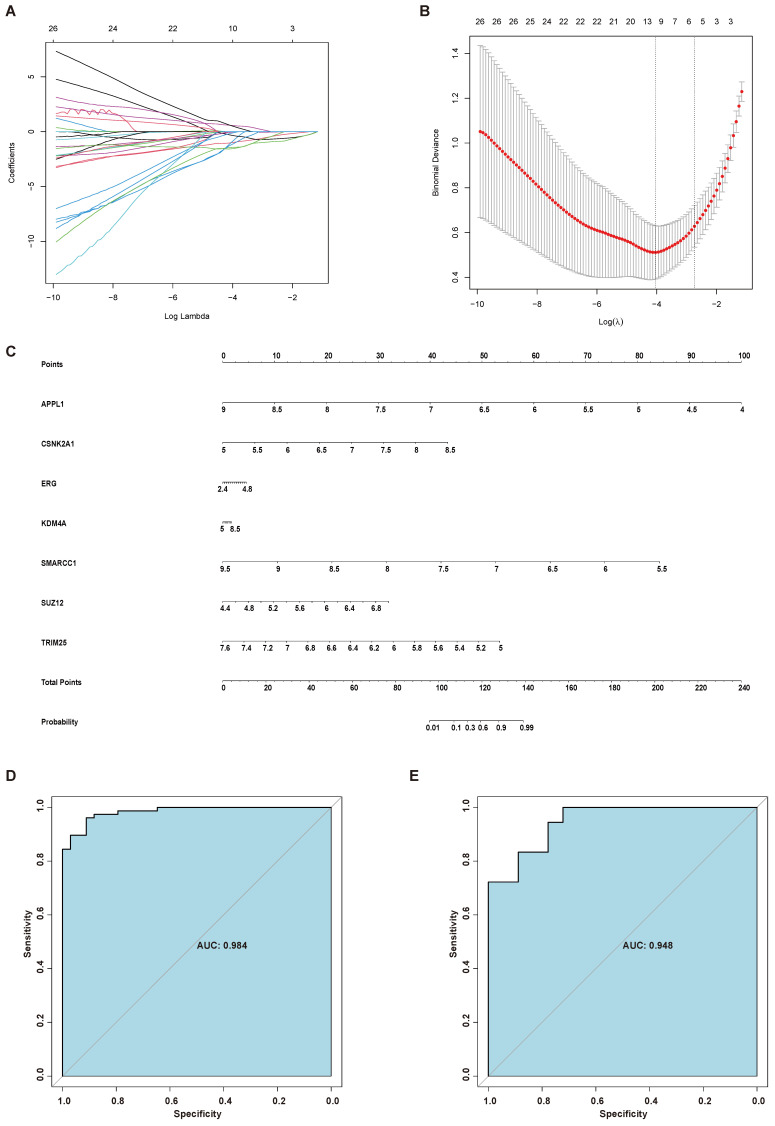
Establishment of a diagnostic model for endometriosis. (A) LASSO coefficient profiles of the genes in the normal endometrium and eutopic endometrium tissue from endometriosis patients. (B) Selection of the optimal parameter (lambda) in the LASSO model for the normal endometrium and eutopic endometrium tissue from endometriosis patients. (C) A nomogram model established based on the LASSO results. (D) ROC curve of the diagnostic nomogram model for the training set (GSE51981). (E) ROC curve of the diagnostic nomogram model for GSE120103. LASSO, least absolute shrinkage and selection operator; ROC curve, receiver operating characteristic curve.

Considering the role of AR in the endometrial aberrations of endometriosis patients, we identified 94 interactors of AR using GPS-Prot and Biogrid. Then, AR and its interactors, including 95 proteins, were further screened by LASSO regression analysis. Seven genes, namely, APPL1, CSNK2A1, ERG, KDM4A, SMARCC1, SUZ12 and TRIM25, were selected to establish the diagnostic model for endometriosis, and a nomogram was constructed based on them (Figure 6A-C). To test the diagnostic efficacy of the nomogram, the ROC curves of the training set GSE51981 and test set GSE120103 were plotted, yielding an AUC of 0.984 for the training set and an AUC of 0.948 for the test set (Figure 6D-E).

## Discussion

Estrogen, progesterone and androgen are well-known hormones that play important roles in female reproductive disease 6, 26-29. The effects of estrogen can be mediated by three types of receptors, including ESR1, estrogen receptor 2 (ESR2) and G-protein coupled estrogen receptor 1 (GPER1/GPR30) 30, 31. Progesterone exerts its biological effects by binding to progesterone receptors, including the nuclear receptor PGR and membrane receptors PGRMC1 and progesterone receptor membrane component 2 (PGRMC2) 32. Upon binding to an androgen, AR can translocate into the cell nucleus and then activate the transcription of its target genes 33.

Previous studies have reported that the decreased ESR1/ESR2 in ectopic lesions leads to the decreased expression of PGR, which can exacerbate the inflammatory response, thereby contributing to endometriosis. In our study, we found that, compared with eutopic endometria, the expression of ESR1 in ectopic lesions was decreased. However, no significant changes in PGR and ESR2 were discovered. The most widely accepted hypothesis for the occurrence of endometriosis is Sampson's retrograde menstruation theory, which postulates that it is retrograde menstruation, which enters the cavity, that results in endometriosis 34. The phenomenon that the prevalence of retrograde menstruation is more than 90% while endometriosis affects only 10% of the female population further reflects that there remain different characteristics between normal endometria and the eutopic endometria of endometriosis patients 34, 35. So, in this study, we also analyzed the expression of the receptors between eutopic endometria of endometriotic patients and normal endometria of healthy women. The results showed that, compared with normal endometria, the expression of AR, PGR, and PGRMC1 in eutopic endometria derived from patients with endometriosis was decreased. Of course, strictly speaking, PGRMC1 doesn't belong to the steroid receptors 36. As a member of a multi-protein progesterone-binding complex, PGRMC1 cannot bind directly to progesterone 36.

Next, the enrichment and PPI analysis were performed. The results showed that the DEGs involved in the eutopic endometrium aberrations of endometriotic patients and ectopic lesions functioned differently. However, both contained genes that participate in the hormone response, in which the nuclear receptors (AR, PGR and ESR1) were included. These nuclear receptors (AR, PGR and ESR1) were also involved in almost all the top 20 enriched biological processes of the DEGs. What's more, both AR and PGR were identified as the hub genes between normal endometria and those of endometriosis patients, and ESR1 was selected as the hub gene between eutopic and ectopic endometria from the same patients. These results imply that sex steroid hormones and their receptors may play important roles in endometriosis development. Besides steroid hormone response, we found that peptide-related processes were also involved in endometriosis development. The synthesis of sex steroid hormones begins with the secretion of gonadotropin-releasing hormone (GnRH), which belongs to peptide hormones 37. In addition, luteinizing hormone (LH), which can stimulate the production of sex hormones, also contributes to the formation of ectopic lesions. Both 'cellular response to luteinizing hormone stimulus' and 'response to luteinizing hormone' were enriched.

Several studies have previously reported the aberrations of estrogen and progesterone receptor pathways in endometriosis 6, 31, 38, 39. As for AR, it is reported that the positive staining of AR can be detected in the stroma and glandular epithelium of eutopic endometrium and ectopic lesions 40, and cytosine, adenine, and guanine (CAG) repeat variants of AR gene were associated with the increased risk of endometriosis 41, 42. However, the aberrations of AR expression in eutopic and ectopic endometrium was uncertain 40. In this study, the IHC results displayed the significantly decreased expression of AR in the eutopic endometrium of endometriotic patients compared with normal endometrium. This study also found a high expression level of AR in the organs of the male and female reproductive systems, such as testis, endometrium and breast. And the positive expression of AR in the main cell types for endometriosis development can also be detected, especially in endometrial stromal cells. However, no significant difference was found between ectopic lesions and their matched eutopic tissues, although the expression of AR in ectopic lesions seemed to be higher. For postmenopausal endometrium, AR expression seemed to be decreased, however, a significant difference has not been detected, possibly due to the limited sample numbers.

Then we performed disease prediction using AR and its interactors. Seven independent factors, including APPL1, CSNK2A1, ERG, KDM4A, SMARCC1, SUZ12 and TRIM25, were filtered. The results showed that the AUC of the nomogram model for the training set (GSE51981) was 0.984; this finding was further verified on the test set (GS120103), with an AUC of 0.948. APPL1 may function as an adaptor protein in many pathways, including the insulin and adiponectin signaling pathways, and suppresses androgen receptor transactivation by potentiating Akt activity 43, 44. APPL1, Akt, and AR form a complex in which Akt serves as the bridge factor for the association of APPL with AR 43. CSNK2A1 is the gene encoding CK2 alpha, the catalytic subunit of protein kinase casein kinase 2 (CK2) 45. CK2 can increase AR protein stability and promote AR-dependent transcriptional activity 45. Additionally, a significant positive correlation was observed between CSNK2A1 and AR mRNA levels in prostate cancer 46. ERG is a member of the E-26 transformation-specific (ETS) family, which has been extensively studied in the field of prostate cancer in recent years 47. ERG can disrupt AR signaling by inhibiting AR expression or by binding to AR at gene-specific loci and inhibiting its activity 48. KDM4A is a histone demethylase related to AR 49. KDM4A can enhance AR-activated gene transcription by forming complexes with ligand-bound AR, thereby mediating multiple processes, including cell proliferation, differentiation, development, and metabolism 50. SMARCC1 is a core subunit of the SWI/SNF complex and has been found to play important roles in the development of several cancers 51, 52. The SWI/SNF complex, containing 5 core subunits and 7-15 accessory subunits, functions by interfering with histone-DNA contacts 53. Almost 25% of all cancers harbor mutations in one or more of these subunits 54. The interaction of SMARCC1 and AR has been shown by affinity capture-MS and affinity capture-western experimental techniques 55, 56. SUZ12 is the core subunit of polycomb repressive complex 2 (PRC2), the epigenetic repressor complex 57. It was reported that PRC2 can regulate the AR-associated signaling pathway 58. The expression of SUZ12 was also correlated with the transcriptional function of AR 59. TRIM25 has been defined as the downstream target of ESR1 and has been shown by affinity capture-MS to interact with AR 59, 60. Although we identified the gene sets that appear to have predictive value for endometriosis development, their use for clinical prediction still needs substantial clinical validation. Instead, the gene sets that can modulate AR signaling were involved in endometriosis development and displayed good predictive value, which also indicates the importance of AR signaling on disease occurrence and provides new targets for the disease. Androgen can inhibit endometrial growth, reduce the chronic pain and inflammation 7, 61, 62. The administration of the synthetic androgen Danazol is effective in treating pain and reducing lesions in endometriosis, but its significant androgenic side-effects limit its use 61. The search for the specific targets of AR signaling regulation in endometriosis may provide the new insight for the development of treatment options. Of course, the further research on the roles of AR in endometriosis development and how these genes influence AR signaling in endometriosis still needs to be further explored.

In summary, this study explored the importance of sex hormone receptors in endometriosis development and improved our understandings of the pathogenesis of endometriosis. Furthermore, the potential roles of AR in endometriosis development provide us new insights into the disease, which may lead to the development of novel treatment strategies.

## Supplementary Material

Supplementary tables.

## Figures and Tables

**Table 1:** The datasets associated with endometriosis.

Datasets	Platform	Sample size	Sample types
GSE141549	GPL10558 & GPL1336	n=408	Ectopicvs normal endometrium & peritoneum
GSE51981	GPL570	33 vs 77	Eutopic vs normal endometrium
GS135485	GPL21290	52 vs 12	Ectopic vs normal endometrium
GSE120103	GPL6480	18 vs 18	Eutopic vs normal endometrium
GSE37837	GPL6480	18 vs 18	Ectopic vs eutopic endometrium
GSE25628	GPL571	n=22	Ectopic vs eutopic vs normal endometrium
GSE5108	GPL2895	11 vs 11	Ectopic vs eutopic endometrium
GSE11691	GPL96	9 vs 9	Ectopic vs eutopic endometrium
GSE99949	GSE17301	4 vs 4	Ectopic vs eutopic endometrium
GSE153740	GPL18573	4 vs 4	Eutopic vs normal endometrium
GSE7305	GPL570	10 vs 10	Ectopic vs eutopic endometrium
GSE58178	GPL6947	6 vs 6	Stromal cells derived from eutopic vs normal endometrium
GSE12768	GPL7304	2 vs 2	Ectopic vs eutopic endometrium

**Table 2:** The enriched biological processes of the DEGs between eutopic and normal endometrium.

	All DEGs	Up-regulated DEGs	Down-regulated DEGs
**Biological process**	Positive regulation of macromolecule metabolic process; Positive regulation of metabolic process; Positive regulation of macromolecule biosynthetic process; Developmental process; Cellular component organization; Cellular component organization or biogenesis; Response to organic substance; Positive regulation of cellular biosynthetic process; Positive regulation of biosynthetic process; Positive regulation of cellular metabolic process; Response to oxygen-containing compound; Single-organism cellular process; Positive regulation of nitrogen compound metabolic process; Response to endogenous stimulus; Positive regulation of gene expression; Cellular response to organic substance; Cellular response to chemical stimulus; Regulation of cellular component organization; Single-organism developmental process; Anatomical structure development.	Cell activation; Anatomical structure development; Leukocyte activation; Regulation of developmental process; Developmental process; Response to oxygen-containing compound; Single-organism developmental process; Response to cytokine; Anatomical structure morphogenesis; Positive regulation of developmental process; Cell surface receptor signaling pathway; Positive regulation of metabolic process; Immune system process; Cellular response to organic substance; Lymphocyte activation; Cellular response to chemical stimulus; Leukocyte differentiation; Positive regulation of macromolecule metabolic process; Multicellular organism development; Single-organism process.	Chromosome organization; Cellular component organization or biogenesis; Organelle organization; Cellular component organization; Establishment of protein localization; Intracellular protein transport; Cellular response to stress; Cellular protein localization; Macromolecule localization; Regulation of mRNA metabolic process; Cellular macromolecule localization; RNA localization; Protein localization; Nitrogen compound transport; RNA splicing, via transesterification reactions with bulged; Adenosine as nucleophile; mRNA splicing, via spliceosome; RNA splicing, via transesterification reactions; Protein transport; RNA splicing.

**Table 3:** The enriched biological processes of the DEGs between ectopic and eutopic endometrium.

	All DEGs	Upregulated DEGs	Downregulated DEGs
**Biological process**	Anatomical structure morphogenesis; Anatomical structure development; Single-multicellular organism process; Multicellular organism development; System development; Developmental process; Single-organism developmental process; Animal organ development; Tissue development; Multicellular organismal process; Cell differentiation; Single-organism cellular process; Cellular developmental process; Cell development; Cell proliferation; Single-organism process; Regulation of cell cycle; Regulation of multicellular organismal process; Response to stimulus; Regulation of developmental process.	Regulation of response to stimulus; Regulation of multicellular organismal process; Positive regulation of response to stimulus; Anatomical structure morphogenesis; System development; Regulation of developmental process; Response to stress; Single-multicellular organism process; Response to stimulus; Multicellular organism development; Multicellular organismal process; Developmental process; Anatomical structure development; Response to wounding; Regulation of immune response; Positive regulation of developmental process; Single-organism developmental process; Regulation of multicellular organismal development; Wound healing; Signal transduction.	Regulation of cell cycle; Cell cycle; Cell cycle process; Regulation of cell cycle process; Cell division; Cell development; Single-organism cellular process; Single-organism process; Anatomical structure development; Tissue development; Nuclear division; Regulation of nuclear division; Animal organ development; Mitotic cell cycle; Single-organism developmental process; Microtubule-based process; Developmental process; Organelle fission; Anatomical structure morphogenesis; Multicellular organism development.

**Table 4:** The hormone-related biological processes of the DEGs between eutopic and normal endometrium

Term	P Value	Genes
**Response to hormone**	1.2421475110305147E-8	CDKN1A, CALCOCO1, AHCYL1, FAM107A, NUCKS1, IRS2, CTSV, SOGA1, AQP1, YY1, RBM4, ZFP36, EDNRA, AIFM1, HADH, JAK3, APPL1, SMARCC1, PRKCI, IGFBP5, NCOA4, SORD, CACYBP, FOS, KLF15, SFRP4, AR, SFRP1, CEACAM1, NCOR1, RAB31, NCL, PGR, PLCB1, RHOQ, SLC29A2, RAMP2, RAMP3, PCNA, WBP2, SRC, SRF, GATA6, PTN, PIK3R1, TYMS, HSPD1, LRP6, SOCS3, SLIT3, FYN, RBBP7, ZBTB7B, EIF4E, SH2B2, ABCA2, EGR1, HSPA8, CCL21, IGF2, CDC6, ATP2B1, SMARCA4, NR4A1, REST, SST, GNB1, FOSB, PAM, SLC26A6.
**Cellular response to hormone stimulus**	1.0762613753458778E-6	CALCOCO1, AHCYL1, FAM107A, NUCKS1, IRS2, SOGA1, AQP1, RBM4, ZFP36, EDNRA, AIFM1, JAK3, APPL1, SMARCC1, PRKCI, NCOA4, FOS, AR, SFRP1, CEACAM1, NCOR1, RAB31, NCL, PGR, PLCB1, RHOQ, SLC29A2, RAMP2, RAMP3, WBP2, SRC, GATA6, PIK3R1, SOCS3, SLIT3, FYN, ZBTB7B, EIF4E, SH2B2, HSPA8, IGF2, CDC6, ATP2B1, SMARCA4, NR4A1, REST, SST, GNB1, FOSB, SLC26A6.
**Response to steroid hormone**	8.59418442951083E-5	CDKN1A, RAMP2, CALCOCO1, PCNA, WBP2, SRC, FAM107A, PTN, CTSV, TYMS, HSPD1, AQP1, ZFP36, AIFM1, SLIT3, RBBP7, EIF4E, ABCA2, HSPA8, NCOA4, ATP2B1, FOS, SMARCA4, AR, SFRP1, REST, NCOR1, SST, FOSB, PGR.
**Cellular response to peptide hormone stimulus**	3.59243815958365E-4	AHCYL1, SRC, NUCKS1, IRS2, PIK3R1, SOGA1, RBM4, SOCS3, FYN, ZBTB7B, JAK3, SH2B2, APPL1, SMARCC1, PRKCI, IGF2, CDC6, FOS, NR4A1, CEACAM1, RAB31, NCL, PLCB1, RHOQ, SLC26A6, SLC29A2.
**Response to peptide hormone**	0.0010350945953294228	AHCYL1, SRC, NUCKS1, IRS2, PIK3R1, SOGA1, LRP6, RBM4, SOCS3, FYN, HADH, ZBTB7B, JAK3, SH2B2, APPL1, EGR1, SMARCC1, PRKCI, IGFBP5, IGF2, CACYBP, CDC6, FOS, KLF15, NR4A1, CEACAM1, RAB31, NCL, PLCB1, RHOQ, SLC26A6, SLC29A2.
**Cellular response to steroid hormone stimulus**	0.011514297795136757	HSPA8, CALCOCO1, WBP2, SRC, NCOA4, FAM107A, ATP2B1, SMARCA4, AQP1, AR, ZFP36, SFRP1, REST, NCOR1, AIFM1, PGR.

**Table 5:** The hormone-related biological processes of the DEGs between ectopic and eutopic endometrium.

Term	P Value	Genes
**Cellular response to luteinizing hormone stimulus**	0.005148208184733331	CCNA2, EDNRA.
**Response to luteinizing hormone**	0.006791515814867501	CCNA2, EDNRA, STAR.
**Hormone catabolic process**	0.015342719212104462	MME, DIO2, HSD17B11.
**Response to peptide hormone**	0.016996690262721416	XBP1, LEPROT, IGFBP5, CAV1, RARRES2, GCNT1, CCNA2, SCNN1G, BRIP1, CXCL12, STAR, SCNN1A, PDK4, TIMP1. JAK3
**Response to hormone**	0.021250037906878627	XBP1, LEPROT, IGFBP5, CAV1, RARRES2, GCNT1, FHL2, TRH, FBXO32, ESR1, RXFP1, TGFBR2, FOXP1, CCNA2, SCNN1G, EDNRA, BRIP1, CXCL12, STAR, SCNN1A, TIMP2, PDK4, TIMP1, JAK3.
**Response to growth hormone**	0.02992832174632198	LEPROT, IGFBP5, STAR, JAK3.
**Cellular response to peptide hormone stimulus**	0.03338644004761159	CCNA2, SCNN1G, LEPROT, XBP1, BRIP1, STAR, CAV1, RARRES2, SCNN1A, PDK4, JAK3.
**Hormone metabolic process**	0.03874287787577624	SCARB1, STAR, MME, ALDH1A2, DIO2, UGT2B28, HSD17B11, ESR1, PAPSS2.

**Table 6:** The enriched biological processes that sex hormone receptors involved in.

	AR	PGR	PGRMC1	ESR1
**Biological process**	Positive regulation of macromolecule metabolic process; Positive regulation of metabolic process; Positive regulation of macromolecule biosynthetic process; Developmental process; Cellular component organization; Cellular component organization or biogenesis; Response to organic substance; Positive regulation of cellular biosynthetic process; Positive regulation of biosynthetic process; Positive regulation of cellular metabolic process; Response to oxygen-containing compound; Single-organism cellular process; Positive regulation of nitrogen compound metabolic process; Response to endogenous stimulus; Positive regulation of gene expression; Cellular response to organic substance; Cellular response to chemical stimulus; Regulation of cellular component organization; Single-organism developmental process; Anatomical structure development.	Positive regulation of macromolecule metabolic process; Positive regulation of metabolic process; Positive regulation of macromolecule biosynthetic process; Developmental process; Response to organic substance; Positive regulation of cellular biosynthetic process; Positive regulation of biosynthetic process; Positive regulation of cellular metabolic process; Single-organism cellular process; Positive regulation of nitrogen compound metabolic process; Response to endogenous stimulus; Positive regulation of gene expression; Cellular response to organic substance; Cellular response to chemical stimulus; Single-organism developmental process; Anatomical structure development; Positive regulation of biological process; Positive regulation of cellular process; Cellular response to endogenous stimulus; Positive regulation of nucleobase-containing compound metabolic process.	Single-organism cellular process; Single-organism process; Cellular biosynthetic process; Organic substance biosynthetic process; Biosynthetic process; Cellular metabolic process; Heterocycle biosynthetic process Aromatic compound biosynthetic process; Cellular process; Cellular nitrogen compound biosynthetic process; Organic cyclic compound biosynthetic process; Organic substance metabolic process; Metabolic process; Single-organism metabolic process; Cellular aromatic compound metabolic process.	Anatomical structure morphogenesis; Anatomical structure development; Single-multicellular organism process; Multicellular organism development; System development; Developmental process; Single-organism developmental process; Animal organ development; Tissue development; Multicellular organismal process; Cell differentiation; Single-organism cellular process; Cellular developmental process; Cell development; Cell proliferation; Single-organism process; Regulation of multicellular organismal process; Response to stimulus; Regulation of developmental process; Regulation of multicellular organismal development.
