# Supplementary material for: Bioinformatics-based analysis of the roles of sex hormone receptors in endometriosis development
Source: Int J Med Sci. 2023 Feb 5;20(3):415–28. doi: 10.7150/ijms.79516 (PMC9969500; doi:10.7150/ijms.79516)
Supplement: Supplementary file 1 — Supplementary tables. [file ijmsv20p0415s1.pdf]

Supplementary Table S1 The gene lists of the DEGs

Groups		Gene lists
Eutopic vs normal endometrium	Up-regulated genes	ABCA2,ABCB9,ABCC3,ABCD1,ABHD14B,ABTB1,ACADS,ACBD4,ACOT11,ACRBP,ADAM11,ADAMTS10,ADAMTS15,ADAMTSL4,ADAP1,ALDH3B2,ANAPC2,ANKRD13D,AP2A1,AP5B1,APOD,AQP1,ARHGAP27,ARHGAP30,ARHGEF1,ATAD3B,ATOH8,ATP13A2,B3GNT7,BCL2L1,BGN,C10orf10,C10orf54,C19orf66,C1orf210,C1QTNF1,C5orf56,CALCOCO1,CBX4,CC2D1A,CCDC184,CCL21,CCL3L3,CCM2L,CD3E,CD7,CD79B,CDA,CDKN1A,CEACAM1,CEBPB,CHPF,CHRD,CLCF1,CLTB,CMTM7,CNTROB,COL5A3,COMP,CPLX1,CPTP,CRTC1,CSF3R,CST1,CTSW,CX3CL1,CXCL2,CYR61,DCAF15,DHX34,DIO3OS,DLGAP1,DMPK,DOK2,DUOX1,DUSP2,EEFSEC,EFHD1,EGFL8,EGR1,EGR3,ELN,EPN1,EPS15L1,ESAM,FAM107A,FAM131A,FAM132A,FAM134A,FAM27E2,FAM27E3,FAM3A,FAM43A,FAM83F,FBLN1,FBXL16,FBXL18,FGD2,FGD3,FGFR1,FGR,FIZ1,FLAD1,FLT3LG,FLYWCH1,FMNL1,FOS,FOSB,FOXP4,FSTL3,FURIN,FUT2,FXYD2,FYN,FZD10AS1,GADD45B,GATA6,GDPD5,GGT3P,GGT5,GGTLC2,GIGYF1,GNA15,GNAO1,GPR157,GPR4,GPX3,GTPBP2,HAB1,HABP2,HAMP,HEMK1,HHLA3,HIC2,HIF3A,HMGA1,HNF1B,HSD11B1L,HSD17B1,HSD3B7,HSPB6,HSPG2,IDS,IDUA,IGFBP5,IGH,IGHA1,IGHA2,IGK,IL2RG,IRS2,IRX3,JAK3,JOSD2,JUNB,KANK3,KAT5,KCNG1,KCNK3,KCTD13,KDM6B,KIR2DL2,KIR2DS2,KIR3DL1,KIR3DL2,KLF15,KLF2,KLF3,KMT5C,KRT18,LDOC1,LENG1,LIF,LILRB3,LOC100289058,LOC100505984,LOC114224,LRCH3,LSS,LY6G5C,LYL1,MAGIX,MAP4K2,MAPK3,MAPK8IP2,MAPRE3,MATN2,MAZ,MBOAT7,MEF2D,MEIS3,MELTF,MID1IP1,MOCS1,MROH1,MROH6,MT1E,MT1G,MTG2,MTSS1L,MUC20,MUSTN1,MVB12A,MYADM,MYO1F,NAA38,NATD1,NDUFA4L2,NEAT1,NEDD4L,NFATC1,NFKBIB,NPR1,NPTXR,NR4A1,NRROS,ORAI3,OSER1AS1,PARVG,PCAT6,PCDHGA7,PCDHGA9,PCDHGB2,PCDHGC5,PCNX3,PCYT2,PDGFB,PDLIM2,PDLIM7,PDZRN3,PGP,PHC2,PHOSPHO2KLHL23,PLA2G2A,PLBD2,PLK1,PLPPR2,PLVAP,PLXNA3,PNKD,POLR2M,PPP2R2A,PRELP,PROK1,PTGER3,PTOV1AS2,PTPRO,RAB1B,RAB20,RAMP2,RAMP3,RANBP3,RAP1GAP,RAPGEF3,RBPMS,RCE1,RDH5,REM1,RGMA,RHOB,RIN1,RIN3,ROGDI,RORC,RPRM,RSG1,S100A1,S100A3,S1PR2,SBNO2,SCAMP4,SEMA3F,SERPINE1,SF3B4,SFTA2,SFXN3,SGK494,SH2B2,SHB,SIX5,SLA2,SLC12A9,SLC25A35,SLC26A6,SLC29A2,SLC30A2,SLC6A16,SLC7A5,SLCO4A1,SLIT3,SMDT1,SMIM24,SMN2,SMTN,SNX21,SNX29,SOCS3,SOGA1,SOX12,SPATA2,SPDYE2,SPHK1,SPINK2,SPNS1,SPSB3,SRC,SRF,SSC5D,SST,ST3GAL2,STAG3L3,STARD10,STARD5,SUN1,SYDE1,SYNGR1,SYNPO,TAF6L,TBC1D17,TBC1D25,TLN1,TM6SF2,TMC8,TMEM86A,TMUB1,TNK1,TNK2,TNPO2,TNS1,TOLLIP,TPSAB1,TRAF7,TRIM52AS1,TRMT2A,TRNP1,TSTD2,TTYH2,UBXN11,UCKL1,UNC13D,VAC14,VMP1,WAS,WBP2,WHRN,WIPF1,WWTR1,ZBTB16,ZBTB22,ZBTB7B,ZC3H10,ZCCHC7,ZDHHC14,ZFP36,ZFP41,ZNF160,ZNF213,ZNF254,ZNF574.
	Down-regulated genes	ACVR2B,ADAM12,ADAMTS16,ADH5,AGR2,AHCYL1,AIFM1,ALCAM,ALDH3A2,ALDH6A1,ALG5,ANK3,ANKRD11,ANKRD17,ANO1,ANP32E,AP3D1,AP3M2,APPL1,AR,ARFGAP3,ARIH2,ARPC2,ARPC5,ASPH,ATP1B3,ATP2B1,ATP2C1,ATP5G3,ATP6V1D,ATPAF1,ATRX,BACE2,BBX,BCLAF1,BECN1,BRCC3,BRI3BP,BTG3,BZW1,C11orf58,C12orf65,C1orf109,C1orf174,C4orf3,C6orf62,C6orf89,C9orf3,CACYBP,CADM1,CANX,CAP1,CASP4,CAST,CBWD5,CBX1,CBX3,CCDC117,CCDC50,CCDC6,CCNB2,CCND2,CCT2,CCT3,CCT4,CD164,CDC40,CDC6,CDH11,CENPF,CENPN,CENPW,CEP55,CFI,CGNL1,CHSY1,CKS1B,CLIC5,CMTM6,CNN3,COL14A1,COL4A3BP,COL5A2,COPB2,COX4I1,CPM,CSDE1,CSNK2A1,CTBP2,CTNNB1,CTSC,CTSV,CTSZ,CYB5B,DCAF7,DCBLD2,DDX24,DDX42,DEPDC1,DHX9,DIO2,DNAJB11,DNAJC10,DNAJC3,DNALI1,DYNC1LI2,EDNRA,EEF1A1,EFCAB14,EGFL6,EID1,EIF3A,EIF3L,EIF4B,EIF4E,EIF4G2,EMC7,EML4,ENC1,ERC1,ERP44,EZR,F13A1,F8A1,FAM155A,FAM169A,FAM199X,FAM60A,FAM98A,FANCD2,FBXL17,FBXO21,FBXO9,FEN1,FNDC3B,FOCAD,FOLH1,FOXN3,FSTL1,G3BP1,G3BP2,GALNT12,GALNT4,GAPVD1,GINM1,GLIS3,GMCL1,GNB1,GOLGA7,GOLIM4,GSTM3,GTF2I,H2AFV,H2AFZ,HACD3,HADH,HBA1,HBA2,HIST1H2AM,HIST1H4B,HIST1H4C,HIST1H4D,HIST1H4E,HIST1H4F,HIST1H4H,HIST1H4I,HJURP,HK2,HN1L,HNRNPA0,HNRNPA1,HNRNPA2B1,HNRNPA3,HNRNPC,HNRNPD,HNRNPH1,HNRNPK,HPGD,HS2ST1,HSP90AA1,HSP90AB1,HSP90B1,HSPA5,HSPA8,HSPD1,IGF1,IGF2,IGF2R,IL13RA1,ILF2,ISOC1,ITGB1,ITIH5,ITPRIPL2,KAT14,KDM4A,KDM5A,KDM5B,KIAA0101,KIAA2013,KIF20A,KIF21A,KIF23,KIF26B,KMT2C,KMT2E,KPNA3,KPNB1,KRT17,LAMC2,LARS,LIMCH1,LMNB1,LMO4,PPP1R16A,LPAR3,LPGAT1,LRP12,LRP6,LYPLA1,LZIC,M6PR,MAGT1,MALAT1,MAP2K3,MAP3K1,MAP4,MAPK1,MARCKS,MAT2A,MCM3,MCM4,MCM6,MECOM,MEST,METAP2,METTL21A,METTL2B,MFF,MGA,MIEF1,MMP7,MORF4L1,MPRIP,MRFAP1L1,MRPL15,MRPL19,MRPS25,MSH6,MTDH,MTMR4,MTPN,MYB,MYLIP,MYOF,NAA35,NAB1,NASP,NBPF10,NBPF19,NBPF3,NBPF9,NCAPD2,NCBP2,NCL,NCOA4,NCOR1,NEK2,NFIA,NGRN,NIP7,NOL8,NORAD,NOTCH2,NPAS3,NPEPPS,NPM1,NREP,NUCKS1,NUP107,NUSAP1,ODC1,OIP5,OVGP1,PAICS,PALLD,PAM,PAPOLA,PCNA,PDIA3,PDIA6,PDZD8,PGR,PGRMC1,PIGY,PIK3R1,PKP2,PLA1A,PLCB1,PLSCR3,PNMAL1,PODXL,PPIC,PPP2R2C,PRC1,PRICKLE1,PRKCI,PRPF4,PRPF40A,PSMA4,PSMA5,PSMA6,PSPH,PTN,PTP4A2,PUM2,PWP1,RAB18,RAB2A,RAB31,RAD51,RALBP1,RAN,RBBP4,RBBP7,RBM17,RBM25,RBM3,RBM4,RBM8A,RC3H1,RCAN3,RCN2,REST,RFC1,RGPD5,RHEB,RHOJ,RHOQ,RMI1,RPAIN,RPL35A,RPL37A,RPN2,RPS23,RPS24,RRBP1,RRM1,RRM2,RSL1D1,RUNX1T1,RYK,SAE1,SAP18,SAR1A,SARNP,SBNO1,SCAF11,SDAD1,SDHC,SEL1L,SEL1L3,SEMA3E,SERBP1,SERPINA5,SET,SETD2,SFRP1,SFRP4,SHROOM3,SKI,SKP1,SLBP,SLC16A3,SLC25A36,SLC31A1,SLC47A1,SMAD9,SMARCA4,SMARCC1,SNRPA1,SNRPD1,SORD,SP100,SPATA13,SPOP,SRP72,SRSF1,SRSF3,SRSF7,SSB,STMN1,STX18,STX2,STXBP6,SUCLG2,SUPT16H,SYNCRIP,TARDBP,TCAF1,TCF4,TEAD1,TGOLN2,THRAP3,THSD4,THYN1,TIA1,TIMELESS,TIMM17A,TMA16,TMED2,TMEM106C,TMEM261,TMEM97,TMX4,TOMM20,TOP1,TOP2A,TPR,TPX2,TRIM2,TRIM25,TRIM4,TRMT5,TSPYL5,TTC3,TUG1,TXNDC5,TXNL4A,TYMS,UBE2T,UBE2V1,UBXN4,UBXN6,UCHL1,UHMK1,VANGL1,VCAN,VDAC1,VIM,WASF2,WHSC1L1,WNT5A,WWC1,YIPF6,YWHAB,YWHAZ,YY1,ZBTB38,ZCCHC17,ZDHHC13,ZKSCAN1,ZMYND11,ZNF148,ZNF22,ZNF24,ZNF644,ZNF704,ZWINT.
Ectopic vs eutopic endometrium	Up-regulated genes	ABCA6,ABLIM1,ACP5,ACTA2,ADIRF,AEBP1,ANTXR2,APOE,ARHGAP22,BAMBI,BEX2,C11orf96,C1QB,C1R,C3,CALD1,CAV1,CBS,CCDC3,CCDC50,CD59,CEBPD,CFD,CFH,CLU,COLEC11,CTSF,CXCL12,DACT1,DCN,EFEMP1,EPAS1,ESYT2,FAM162A,FAM46A,FBXO32,FGF7,FGL2,FHL2,FLJ30901,FMOD,FN1,FOXN3,FOXP1,FTL,G0S2,GNG4,GPNMB,HLADPB1,HLADRA,HLADRB1,HLAE,HMOX1,HOPX,HS3ST1,HSD17B11,HSPA12A,IFI16,IGFBP3,IGFBP5,JAK3,JUNB,KLF2,KLHDC8A,LAMB2,LEPROT,LHFP,LMCD1,LXN,MAN1C1,MAST4,MCUB,MEG3,MYL9,NDRG1,NEFH,NUPR1,OPTN,PALM2AKAP2,PAPSS2,PDGFRL,PDK4,PEG3AS1,PLA2G16,PLCXD3,PMP22,PRNP,PROS1,PTGIS,PTRF,RABGAP1L,RARRES2,RARRES3,RASSF3,RGS2,RNASE1,ROBO3,RPL23AP32,RPP25,SCARB1,SEPP1,SGCE,SH3BP5,SH3D19,SLCO2B1,SMOC2,SOBP,SOD3,SP100,ST13,STAR,STON1,TAGLN,TCEAL3,TCEAL4,TGFBR2,THBS2,THBS4,TIMP1,TIMP2,TLCD2,TPM1,TPM2,TRPV2,TSPAN4,UCHL1,VLDLR,VSIG4,VWF,WISP2,WWTR1,ZEB2,ZNF275,ZNF395.
	Down-regulated genes	AADAT,ADAMTS6,ADAMTS9,ADGRG2,AGMAT,AGR3,ALDH1A2,ANO1,APOBEC3B,ARHGAP26,ARSJ,ASPM,ATP5G3,ATP6V1C2,ATP8B3,AURKA,BCAS1,BRI3BP,BRIP1,BTBD11,C1orf168,C2orf88,C4orf19,CAPSL,CASZ1,CBLN1,CCNA1,CCNA2,CCNB1,CDC20,CDCA2,CDCA3,CDKN3,CECR2,CENPF,CENPN,CFAP43,CHEK1,CHMP4C,CITED4,CLIC5,CLMN,COBL,CRISPLD1,CTAGE5,CTNNA2,CXADR,DACH1,DACT2,DEPDC1B,DHFR,DIO2,DLX6,DLX6AS1,DNAJC22,DOK7,DTL,DUSP4,E2F7,E2F8,ECT2,EDNRA,EHF,ELF3,ENPP3,ERBB3,ERICH3,ESR1,ESRP1,EXPH5,FAM169A,FAM83D,FBN2,FEN1,FOLH1B,FOXA2,FRAS1,GCNT1,GCNT2,GJB6,GPR160,GPSM2,GREM2,GTSE1,HELLS,HHIP,HJURP,HOMER2,HOOK1,HOXA11,HOXA5,HOXB8,HOXD11,IL17RB,IRF6,KIF11,KIF14,KIF18A,KIF20A,KIF21A,KIF5A,KLHL13,KMO,KNL1,KRT23,LAMC2,LINC00261,LOC100190986,LOC100506098,MAP3K1,MAP7,MCM10,MCM4,MET,MFAP3L,MFSD2A,MKI67,MLPH,MME,MMP16,MPPED2,MREG,MS4A8,MSX1,MSX2,MTCL1,MTHFD2L,MUC16,NEDD9,NEK2,NRCAM,NREP,NRTN,NRXN1,NUP62CL,NUSAP1,OCLN,OLFM4,ORM1,ORM2,OVOL2,PANK1,PARPBP,PATJ,PBK,PKD1L2,PLCH1,PLPPR4,PLS1,PMAIP1,POLE2,PPP2R2C,PRR15,PRR15L,PRR5ARHGAP8,PRRG4,PRSS16,PRSS8,PTCH1,RAB25,RAD51,RAD51AP1,RASD1,RASEF,RCAN3,RIPK4,RNF183,RORB,RRM2,RXFP1,SCGB1D2,SCNN1A,SCNN1G,SERINC2,SFN,SHANK2,SHISA6,SLAIN1,SLC15A2,SLC26A2,SLC34A2,SLC35A3,SLC44A3,SLC44A4,SLC46A2,SMC2,SMPDL3B,SOX9,SPATA17,SPATA18,SPINT1,SSX2IP,ST6GAL2,STRBP,STXBP6,TACC3,TACSTD2,TBX3,TFCP2L1,TMC4,TNNC1,TPBG,TPD52,TPX2,TRAF3IP2,TRH,TRIP13,TUBB2B,UBXN10,UGT2B28,UGT8,VTCN1,WEE1,WHSC1,XBP1,XPR1,ZBBX,ZCCHC12,ZMYND8,ZNF367.

Supplementary Table S2 The selected hub genes

Groups	Hub genes
Eutopic vs normal endometrium	ABCD1;AGR2;AHCYL1;ALDH3B2;ANKRD17;AR;ATP6V1D;BACE2;BBX;CANX;CCDC117;CENPN;COX4I1;CSNK2A1;CST1;CTNNB1;CTSW;CYB5B;DCBLD2;DDX24;DDX42;DNAJC10;DUSP2;EIF3A;EIF4B;EIF4E;EIF4G2;FGFR1;FYN;G3BP1;GADD45B;GNA15;GNAO1;GTF2I;HIST1H2AM;HIST1H4B;HIST1H4C;HIST1H4D;HIST1H4E;HIST1H4F;HIST1H4H;HIST1H4I;HJURP;HNRNPA0;HNRNPA1;HNRNPA2B1;HNRNPA3;HNRNPC;HNRNPD;HNRNPH1;HNRNPK;HSP90AA1;HSP90AB1;HSP90B1;HSPA5;HSPA8;HSPD1;ITGB1;KCNK3;KDM5A;KDM5B;KDM6B;KIF20A;KIF23;KMT2C;KPNA3;MAGT1;MAP3K1;MAPK1;MAPK3;METTL2B;MORF4L1;MT1E;MT1G;MTPN;NASP;NCBP2;NCL;NFATC1;NOL8;PALLD;PDIA3;PDIA6;PGR;PIK3R1;PPIC;PRC1;PRKCI;PUM2;RAD51;RBBP4;RBM25;RBM3;RBM4;RBM8A;RC3H1;ROGDI;RRBP1;RUNX1T1;RYK;SEMA3E;SEMA3F;SERPINA5;SGK494;SMARCC1;SNRPD1;SOGA1;SRC;SRF;SRSF1;SRSF7;SSB;TARDBP;TIA1;TMUB1;TNK1;TNK2;VIM;WAS;WHSC1L1;YWHAB;YWHAZ;ZFP36.
Ectopic vs eutopic endometrium	ACTA2;ASPM;AURKA;CAV1;CCNA1;CCNA2;CCNB1;CD59;CDC20;CENPF;CLU;COBL;E2F7;E2F8;ECT2;ESR1;ESRP1;FAM83D;HOMER2;KIF14;KIF20A;MAP3K1;MET;NRCAM;NUSAP1;PAPSS2;PLS1;POLE2;PRNP;PTRF;RASEF;RIPK4;RNF183;SFN;SHANK2;STXBP6;TNNC1;TPM1;TPM2;TPX2;TRIP13;TUBB2B.
